# Drought, Smallpox, and Emergence of *Leishmania braziliensis* in Northeastern Brazil

**DOI:** 10.3201/eid1506.071331

**Published:** 2009-06

**Authors:** Anastácio Q. Sousa, Richard Pearson

**Affiliations:** Federal University of Ceará, Fortaleza, Brazil (A.Q. Sousa); University of Virginia, Charlottesville, Virginia, USA (A.Q. Sousa, R. Pearson)

**Keywords:** Drought, smallpox, cutaneous leishmaniasis, Leishmania braziliensis, Brazil, viruses, parasites, historical review

## Abstract

Cutaneous leishmaniasis caused by *Leishmania (Vianna) braziliensis* is a major health problem in the state of Ceará in northeastern Brazil. We propose that the disease emerged as a consequence of the displacement of persons from Ceará to the Amazon region following the Great Drought and smallpox epidemic of 1877–1879. As the economic and social situation in Ceará deteriorated, ≈55,000 residents migrated to the Amazon region to find work, many on rubber plantations. Those that returned likely introduced *L. (V.) brazilensis* into Ceará, where the first cases of cutaneous leishmaniasis were reported early in the 20th century. The absence of an animal reservoir in Ceará, apart from dogs, supports the hypothesis. The spread of HIV/AIDS into the region and the possibility of concurrent cutaneous leishmaniasis raise the possibility of future problems.

## Abstract 2

The Great Drought and smallpox epidemic (1877–1879) led to emergence of L. braziliensis in Ceará State, northeastern Brazil

The emergence of cutaneous leishmaniasis in northeastern Brazil in the state of Ceará illustrates how environmental and human factors combine to influence human health. Cutaneous leishmaniasis is an important health problem for residents of Ceará. In the 20 years from 1986 to 2005, >49,000 new cases were reported (Figure 1) (*1*). Given the difficulties in reporting in rural areas, the true number is likely substantially higher. Today, the disease is distributed across several areas in the state, but this has not always been the case. In fact, historical data suggest that cutaneous leishmaniasis is a relatively recent arrival and may have come from the Amazon region as a consequence of drought, smallpox, and social and economic conditions that led to human migration.

**Figure 1:**
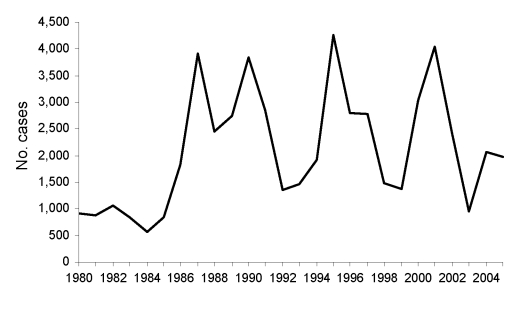
Cases of cutaneous leishmaniasis in the state of Ceará, Brazil, 1980–2005. Source: Ministry of Health, Brazil.

The first well-documented cases of cutaneous leishmaniasis in Ceará date back to 1925 when photographs of patients with classic skin ulcers and advanced mucosal leishmaniasis (Figures 2, 3) were published in a report on endemic diseases in the state (*2*). The author, A. Gaviao-Gonzaga, hypothesized that the disease may have been introduced from the Amazon. Sales (*3*), the first to map the geographic distribution of the disease in the state, also suggested this possibility, but neither report provided specific evidence to support the hypothesis.

**Figure 2:**
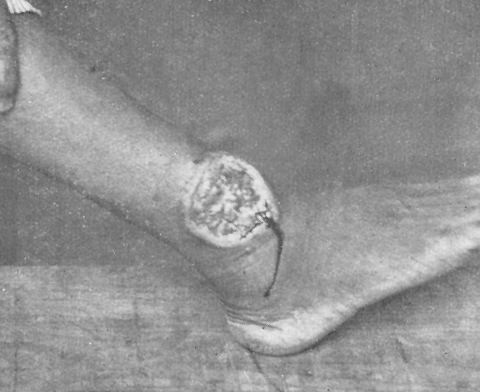
Weeping ulcer (pizza-like lesion) in a patient with typical cutaneous leishmaniasis, Ceará, Brazil. Source (*2*).

**Figure 3:**
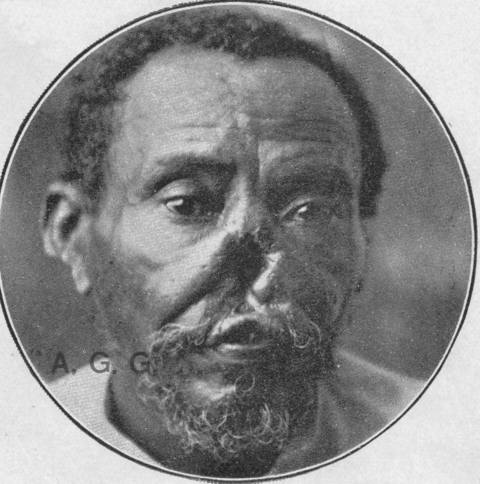
Advanced mucosal leishmaniasis in a patient who had cutaneous leishmaniasis, Ceará, Brazil. Source (*2*).

We investigated the possible origin of cutaneous leishmaniasis in Ceará and postulate that the disease emerged as a consequence of the displacement of >55,000 persons from Ceará to the Amazon region at the end of the 1800s (*4*) after the Great Drought of the 1870s (*5**,**6*) and an unprecedented epidemic of smallpox that left a death toll of >100,000 persons (*7**–**9*). We propose that migrants became infected in the Amazon and carried the disease with them when they returned to Ceará and elsewhere in eastern Brazil. As a result, the disease was introduced into northeastern Brazil and subsequently emerged as an important health problem.

## The Great Drought

Drought is a natural phenomenon that has afflicted the lives of millions of persons over the centuries and remains an important cause of human illness in many regions of the globe. Its greatest effects frequently occur in developing areas with agrarian societies and few natural resources. Depending on the region, drought can cause serious problems in humans through famine and later malnutrition, in addition to the death of livestock, alteration of the area’s wildlife, and acceleration of deforestation. Drought frequently stimulates migration and displacement of large groups of people. These events are well documented in the history of northeastern Brazil.

The state of Ceará in northeastern Brazil is one of the poorest in the country in respect to natural resources. Its population is >8 million inhabitants, representing 4.2% of the country’s population. Ceará is known as the “land of light.” Many attribute the saying to its hot, sunny, and dry weather that occurs almost year round; the real reason is that it was the first state in Brazil to abolish slavery. Unfortunately, droughts have occurred periodically in the region for as long as records have been kept. A list of the droughts since the beginning of the 17th century is available, but it is no doubt incomplete because less severe droughts were not registered (*9*). Studart, in discussing the history of drought in Ceará, wrote the following: “If Ceará was a land of regular rains and well distributed, in Brazil no state would compete with it; however, the drought that persecutes it, impairs its flight to incomparable destinies” (*9*).

The first severe drought in Ceará in the 19th century occurred in 1824. A second drought followed in 1844. Because 2 decades had elapsed between those droughts, it was thought that there was a 20-year drought cycle. When no drought occurred in 1864–65, many thought Ceará was free of them (*4**,**9*). Rapid development followed.. Cotton exports to the United States were increased because of the US Civil War. These exports to the United States decreased after the war, and exports also decreased to England after incentives were provided for cotton plantations in some of England’s colonies (*4*). However, the agricultural economy remained strong because ample rainfall from 1870 to 1876 enhanced the production of other crops. By 1872, Ceará had a population of 721,686 persons, and Fortaleza, its capital city, had grown to 23,500 (*6*).

The Great Drought, the most severe ever recorded in Brazil, began in Ceará in 1877 and lasted 3 years. Drought conditions also occurred in several other northeastern states (*5*), but Ceará was by far the most severely affected. The delayed and inadequate response of the central Brazilian government exacerbated the problems (*8*).

As the drought continued, the number of persons needing assistance grew quickly and by 1878 had reached 480,000. Those persons in rural areas in the central part of the state migrated to the capital and other cities in mountainous areas and along the coast where the effects of the drought were less severe. Fortaleza was in a state of calamity. As persons arrived in the city, they lived on the outskirts of the city in inhumane conditions with very little food and or sanitation. The situation was ripe for disaster, and it happened.

## The Smallpox Epidemic

Smallpox was beyond a doubt one of the greatest scourges of humanity. It was responsible for much human illness, millions of deaths over centuries, and the elimination of entire societies, for example, the Mississippian chiefdoms in North America between 1491 and the late 1600s (*10**,**11*).

In September 1878, smallpox arrived in Ceará, brought by a sailor from a ship that had anchored at a coastal city near Fortaleza (*8*). The disease quickly reached Fortaleza and spread to other cities. An estimated 95% of the population in Fortaleza and ≈100% of those living in the other cities had not been vaccinated and were susceptible to infection (*8*). Theophilo (*8*) wrote that the disease spread like a fire in dried leaves fanned by a strong wind.

**Figure 4:**
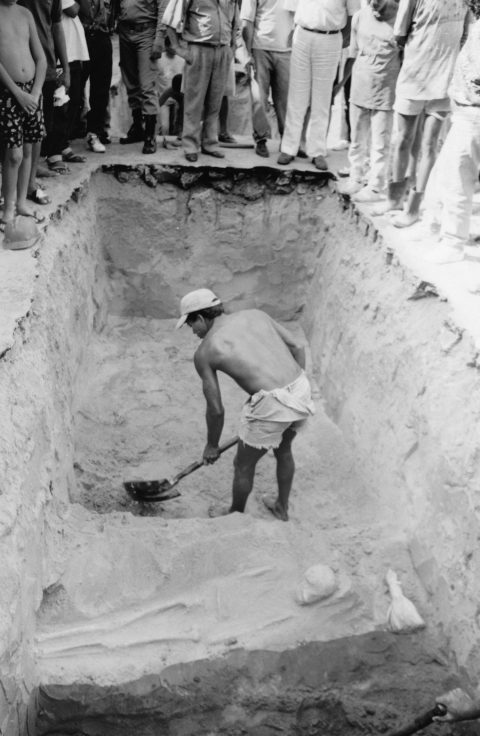
Discovery of human skeletons during excavation in 1994 of new sewage system in Ceará, Brazil, of persons who died of smallpox during the epidemic of 1877–1879. Photo courtesy of Jornal O Povo.

In Fortaleza, 62 people died of smallpox in September 1878. In October, the number had risen to 592 and in November to 9,844. In December, 15,435 persons died, >500 deaths per day. December 10, 1878, was the saddest day of all; 1,004 persons died of smallpox in Fortaleza alone. Many were left unburied because of the lack of healthy persons to bury them. This tragedy was brought home in a poignant way in 1994 when excavation for a new sewage system unearthed skeletons in shallow graves (Figure 4) in Fortaleza believed to have been buried on the “day of one thousand deaths” (*12*).

Thirty years later, Studart (*9*), who had only recently graduated from medical school when the drought began, recalled the horror:

“The year of 1878 started, and with it grew to the infinite the anguish of the people from Ceará. People died of hunger, purely of hunger on the streets and on the roads, after they had eaten roots of many different plants. The starving people ate the most repugnant meat; reptiles, dogs, vultures and crows. Although rare, cases of anthropophagy occurred; individuals were seen eating human flesh. Bodies were found with part of the limbs partially eaten due to the extreme despair of human famine. … December 10 [1878], I remember that horrific day; 1,004 people died of smallpox in Fortaleza. They were brought to the cemetery and many were not buried due to the tiredness of the buriers. It is registered that an average of 500 individuals died a day; however, the numbers must have been much higher, because around the city, into the bushes and into closed houses where whole families had died, bodies were found in a state of putrefaction.”

**Figure 5:**
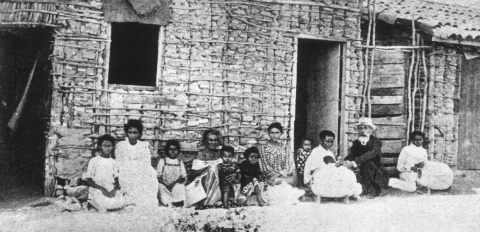
Rodolpho Theophilo (second from right) during a smallpox vaccination campaign he led during 1901. Photo courtesy of Nirez Archives.

Rodolpho Theophilo (*12*), a local pharmacist, began producing the smallpox vaccine in a laboratory he had built with his own money because the vaccine from Rio de Janeiro was not apparently protective. He personally vaccinated thousands of persons over a 4-year period (Figure 5) and created a chain of volunteers in other cities in the state to whom he sent the vaccine with a packet insert containing instructions. He recalls the situation (*8*):

“The excessive heat of 33 degrees centigrade in the shade in that fatal December added to the epidemic. There was total disorientation. In the tenth of the month the cemetery received 1,004 dead bodies, that horrifying obituary of just one day, let all those who received the news in panic. … At the end of the day 230 dead bodies were left unburied. … In the next morning when the buriers returned to continue their work, they found dogs and vultures feeding on human bodies.”

News of the terrible calamity reached the New York Herald, the Medical Times and Gazette of London, and the New York Times (*6*). Articles in the New York Times appeared on November 17, 1877, and on February 24, 1879. The latter article, entitled “Pestilence and famine in Brazil,” described the disaster (*7*): “During the 3 years (1877–1879) of drought, 150,000 persons died in Ceará state, most of them in 1878 (118,927 deaths). In Fortaleza, 67,267 deaths occurred in the 3-year-period, 57,780 of those in 1878.”

As the economic and social situation in Ceará worsened in 1878, a call went out for workers in the Amazon region where rubber production was rapidly developing. In that year, 54,875 (*4*) persons migrated to the Amazon. The migration to the Amazon occurred in the period known as the rubber boom (*13*), several decades after the discovery of vulcanization by Charles Goodyear in 1839. In the following years, many more people went to the Amazon in pursuit of better living conditions. Many returned to their families in Ceará; others died of malaria, a frequent cause of death in the Amazon in those days (*14*).

## The Emergence of Cutaneous Leishmaniasis in Ceará

Archeologic evidence suggests that *Leishmania* species that cause cutaneous leishmaniasis were present in South America long before the arrival of Europeans. Human disease has been recognized in Peru since Inca and pre-Inca times. Facial mutilations consistent with mucosal leishmaniasis have been observed in Peruvian pottery images (*15*). Written description of the Peruvian form of leishmaniasis called *uta* dates back to 1764, when the disease was already endemic in many areas of the country (*16*).

Although human leishmaniasis was known in Peru in antiquity, it has been recognized in Brazil for little more than a century. The first clear clinical description of cutaneous leishmaniasis in Brazil was made in 1895 in Bahia (*17*); however, Rabello (*18*) cites a report of a missionary trip in the Amazon region in a publication dated 1827, which noted that it was common to see persons with ulcers in their arms and legs as well as destructives lesions around the mouth and nose and that those were caused by mosquito bites. The descriptions are consistent with leishmaniasis (*18*).

We have been unable to find any reference to cutaneous or mucosal disease consistent with *Leishmania braziliensis* infection in Ceará in a careful review of government documents, books and newspapers from 1830 until Studart’s 1909 report (*9*) of a skin condition that might have been leishmaniasis. The first official reference to cutaneous leishmaniasis appeared in a 1917 government report (Public Library, Ceará 1917). In 1912, Gaspar Vianna (*19*), who discovered trivalent antimony treatment for cutaneous leishmaniasis, reported treating patients from many states in Brazil, including Ceara (*20*). Accounts of the first well-documented autochthonous cases in Ceará were published in 1925 with photographs of persons with cutaneous and mucosal lesions (*2*). An alternative hypothesis proposed that *L. braziliensis* was present in animals before the Great Drought and smallpox epidemic of the 1870s, but the lack of early reports suggests that this was not the case, and even today no animal reservoir other than dogs has been identified in Ceará.

Cutaneous leishmaniasis is currently endemic in a number of areas of Ceará. Most are located in mountainous regions and in areas adjacent to the coast where people immigrated during the Great Drought. Although the disease may have previously existed there, and healthcare workers failed to observe or report the chronic skin and mucosal lesions, we believe that it is more likely that persons who had immigrated to the rubber plantations in the Amazon after the Great Drought and smallpox epidemic brought *L. braziliensis* infection to Ceará, either through human or animal infection. Several observations support this finding.

Considering that leishmaniasis was known to exist in Peru for centuries, why it did not emerge in Brazil earlier? The reason is not totally clear. The Incas did not settle in the Amazon Basin, presumably for economic reasons and due to their preference for vertical landscapes (*21*). The disease may well have been present for many years among Indian tribes in the Amazon region, but they had little communication and interaction with the rest of the country until the start of the rubber industry, which intensified after vulcanization was discovered.

The parasite was first identified in 1909. Lindenberg (*22*) and Carini and Paranhos (*23*), working independently, identified the protozoan during an epidemic of “ulcera de Bauru” or Bauru sore that accompanied the construction of a railroad between the cities of Bauru in São Paulo and Corumba in Mato Grosso states. The name *L. braziliensis* was given by Vianna in 1911 (*24*). He observed the parasite in smears from a person with disseminated cutaneous leishmaniasis, an uncommon manifestation of infection (*25*). Vianna, in examining smears from the lesions, described the morphologic features of the parasite, including the kinetoplast and a thin linear structure that is not seen with currently used Wright and Wright-Giemsa stains. Vianna concluded that he was dealing with a new species of *Leishmania.* It is likely that the linear structure was the inner lying flagellum, which is easily seen by electron microscopy (*26*). d’Utra e Silva (*20*), who worked with Vianna, explained the staining methods.

*L.* (*Viannia*) *braziliensis* has the widest geographic distribution of the *Leishmania* species endemic in Latin America. It has been documented to cause human disease in 14 countries (*27*). In Brazil, it is responsible for most cases of leishmaniasis (*28*), and in Ceará, 272 isolates from patients with cutaneous leishmaniasis, representing all disease-endemic areas, were characterized as *L. braziliensis* (*29*).

The spectrum of disease caused by *L. braziliensis* is broad. It includes an early lymphadenophathic form, the classic weeping cutaneous ulcers, disseminated cutaneous leishmaniasis, and mucosal disease (*25*,*29*,*30*). Mucosal involvement may occur simultaneously with the cutaneous lesion(s), but most cases are diagnosed months to years after the cutaneous ulcer has healed (*31*).

## Leishmaniasis in the 21st Century

**Figure 6:**
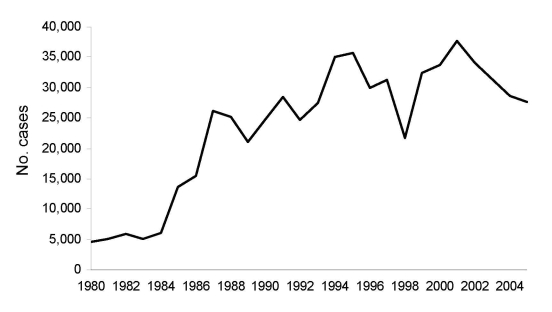
Cases of cutaneous leishmaniasis, Brazil, 1980–2005. Source: Ministry of Health, Brazil.

Much has transpired in northeastern Brazil since the Great Drought and smallpox epidemic of the 1870s. Cutaneous leishmaniasis has grown into a major health problem in the region and across Brazil. The total number of cases reported in the country from 1980 to 2005, was 613,644 (Figure 6). At least 9 Brazilian states now have >1,000 cases each year. Ceará is among the top 5 states, and in some years, it ranks first in the total number of cases (*1*).

Although smallpox has been eradicated, HIV infection has emerged. There is great concern that concurrent infection with HIV and *Leishmania* may pose major problems in the future (*32*). It appears that each infection can worsen the course of the other, acting in synergistic ways to shorten the incubation periods and increase progression of both (*33*). The incidence of HIV/AIDS is increasing in northeastern Brazil, and HIV is extending into rural areas where the prevalence of cutaneous leishmaniasis is high (*34*).

It is important to note that persons who are infected with *Leishmania* species, with or without symptoms, appear to remain infected. Reactivation can occur if cellular immunity diminishes as a result of HIV. Identifying co-infected persons will be crucial so that appropriate highly active antiretroviral therapy and antileishmanial therapy can be initiated. The skin load of *Leishmania* species is much higher in HIV-infected persons, and although not proven, these patients may be a source of infection for the sand flies in disease-endemic areas.

For decades, the treatment of *L. braziliensis* infection has been based on parenteral administration of the pentavalent antimony. The drug is toxic and variably effective. A more effective, safer, cheaper, and, preferably, oral alterative is badly needed. Some progress has been made in recent years in new drug development (*35*,*36*). Prospects for an effective vaccine in the near future seem limited. Finally, a sylvatic reservoir has not been identified for *L. braziliensis* in Ceará and other areas. Dogs appear to be the most important reservoir in domestic and peridomestic transmission (*37*). Preliminary data on the use of deltamethrin-impregnated dog collars appear promising (*38*), but additional studies and government sponsorship are needed if they are to be widely implemented. Advances in one or more of these areas are essential to reverse the effects of *L. braziliensis* in the region and elsewhere.

## References

[1] Ministry of Health. Brazil [cited 2007 Jul 29]. Available from http://portal.saude.gov.br/portal/arquivos/pdf/leishmaniose_2006.pdf

[2] Gaviao-Gonzaga A. Climatologia e nosologia do Ceará, paginas de medicina tropical. Rio de Janeiro (Brazil): Batista de Sousa; 1925.

[3] Sales JB. Geografia médica do Ceará: distribuição geográfica da leishmaniose. Rev Bras Med. 1952;9:496–8. 13064477

[4] Pinheiro FJ. Ceara: Seca e Migracao. In: Silva SV, editor. A igreja e a questao agraria no nordeste: subsidios historicos. São Paulo (Brazil): Edicoes Paulinas; 1986. p 31–49.

[5] Greenfield GM. The realities of images, imperial Brazil and the Great Drought. Trans Am Philosophical Soc, New Series, 2001;91:i–xxvi+1–148.

[6] Costa MCL. Teorias médicas e gestão urbana: a seca de 1877–79 em Fortaleza. Hist Cienc Saude Manguinhos. 2004;11:57–74. 10.1590/S0104-59702004000100004 15318399

[7] Jackson WR. Pestilence and famine in Brazil. NY Times. 1879 Feb:24.

[8] Theophilo R. Variola e vacinacao no Ceará. Fortaleza (Brazil): Officinas typographicas do Jornal do Ceara; 1904.

[9] Studart G. Climatologia, epidemias e endemias do Ceará. Fortaleza (Brazil): Typographya Minerva; 1909.

[10] Hopkins DR. The greatest killer, smallpox in history. Chicago: University of Chicago Press; 2002.

[11] Diamond J. Guns, germs, and steel, the fates of human societies. New York: W.W. Norton; 2005.

[12] Lira Neto. O poder e a peste: a vida de Rodolfo Teófilo. Fortaleza (Brazil): Edições fundação Demócrito Rocha; 1999.

[13] Aitchison M. The tree that weeps: a history of Amazon rubber. Letter from Manaus [cited 2007 Jul 4]. Available from http://www.brazilmax.com/columnist.cfm/idcolumn/38

[14] Chagas C. Nota sobre a epidemiologia do Amazonas. Bras Med. 1913;27:450–6.

[15] Herrer A. Antiguedad de la leishmaniasis tegumentaria en America. Rev Bras Malariol Doencas Trop. 1956;8:187–93. 13494839

[16] Herrer A, Christensen HA. Implication of *Phlebotomus* sand flies as vectors of bartonellosis and leishmaniasis as early as 1764. Science. 1975;190:154–5. 10.1126/science.1101379 1101379

[17] Moreira J. Existe na Bahia o Botao de Biskra? Estudo Clinico. Gazeta Médica da Bahia. 1995;254–8.

[18] Rabello E. Contribuição ao estudo da leishmaniose tegumentar no Brasil. Origens, histórico e synonimia. Annaes Brasileiros de Dermatologia e Syphilographia. 1925;1:3–31.

[19] Vianna G. tratamento da leishmaniose tegumentar por injeções intravenosas de tártaro emético. Arq Bras Med. 1912;4:426–8.

[20] de’Utra e Silva A. Sobre a leishmaniose tegumentar e seu tratamento. Mem Inst Oswaldo Cruz. 1915;7:213–48.

[21] Le Moine G, Raymond JS. Leishmaniasis and Inca settlement in the Peruvian jungle. J Hist Geogr. 1987;13:113–29. 10.1016/S0305-7488(87)80142-1

[22] Lindenberg A. L’ulcère de Bauru ou le bouton d’Orient au Brésil. Communication préliminaire. Bull Soc Pathol Exot. 1909;2:252–4.

[23] Carini A, Paranhos U. Identification de l’Ulcera de Bauru avec le bouton d’Orient. Bull Soc Pathol Exot. 1909;2:255–7.

[24] Vianna G. Sobre uma nova espécie de leishmania. Brazil Medico. 1911;25:411–112.

[25] Turetz ML, Machado PR, Ko AI, Alves F, Bittencourt A, Almeida RP, Disseminated leishmaniasis: a new and emerging form of leishmaniasis observed in northeastern Brazil. J Infect Dis. 2002;186:1829–34. 10.1086/345772 12447770

[26] Gull K. The cytoskeleton of Trypanosomatid parasites. Annu Rev Microbiol. 1999;53:629–55. 10.1146/annurev.micro.53.1.629 10547703

[27] Shaw JJ. New World leishmaniasis: The ecology of leishmaniasis and the diversity of leishmanial species in Central and South America. In: Farrell J, editor. World class parasites, vol 4. *Leishmania*. Boston: Kluwer Academic Publishers; 2000. p. 9–31.

[28] Lainson R. The American leishmaniasis: some observation on their ecology and epidemiology. Trans R Soc Trop Med Hyg. 1983;77:569–96. 10.1016/0035-9203(83)90185-2 6197791

[29] Sousa AQ, Parise ME, Pompeu MM, Coelho Filho JM, Vasconcelos IA, Lima JW, Bubonic leishmaniasis: A common manifestation of *Leishmania (Viannia) braziliensis* infection in Ceara, Brazil. Am J Trop Med Hyg. 1995;53:380–5. 7485690 10.4269/ajtmh.1995.53.380

[30] Pearson RD, Sousa AQ. Clinical spectrum of leishmaniasis. Clin Infect Dis. 1996;22:1–13. 8824958 10.1093/clinids/22.1.1

[31] Marsden PD. Mucosal leishmaniasis (“espundia,” Escomel, 1911). Trans R Soc Trop Med Hyg. 1986;80:859–76. 10.1016/0035-9203(86)90243-9 3037735

[32] Rabello A, Orsini M, Disch J. Leishmania/HIV co-infection in Brazil: an appraisal. Ann Trop Med Parasitol. 2003;97(Suppl.1):S17–28. 10.1179/000349803225002507 14678630

[33] Wolday D, Berhe N, Akuffo H, Britton S. Leishmania-HIV interaction: immunopathogenic mechanisms. Parasitol Today. 1999;15:182–6. 10.1016/S0169-4758(99)01431-3 10322351

[34] Ministry of Health. Brazil [cited 2007 Aug 17]. Available from http://www.aids.gov.br/data/documents/storedDocuments/%7BB8EF5DAF-23AE-4891-AD36-1903553A3174%7D/%7B6B12D137-92DF-4CF5-A35A-482AED64CBC0%7D/BOLETIM2006internet.pdf

[35] Croft SL, Seifert K, Yardley V. Current scenario of drug development for leishmaniasis. Indian J Med Res. 2006;123:399–410. 16778319

[36] Mishra J, Saxena A, Singh S. Chemotherapy of leishmaniasis: past, present and future. Curr Med Chem. 2007;14:1153–69. 10.2174/092986707780362862 17456028

[37] Oliveira-Lima JW. Domestic transmission of cutaneous leishmaniasis in Brazil [doctoral dissertation]. Cambridge (MA): Harvard University; 1995.

[38] David JR, Stamm LM, Bezerra HS, Souza RN, Killick-Kendrick R, Lima JW. Deltamethrin-impregnated dog collars have a potent anti-feeding and insecticidal effect on *Lutzomyia longipalpis* and *Lutzomyia migonei.* Mem Inst Oswaldo Cruz. 2001;96:839–47. 10.1590/S0074-02762001000600018 11562713

